# 1364. Association Between Tixagevimab/Cilgavimab Administration and Self-Reported SARS-CoV-2 Infection During the Omicron Surge

**DOI:** 10.1093/ofid/ofad500.1201

**Published:** 2023-11-27

**Authors:** Daniya S Mathew, Patricia Saunders-Hao, Sumeet Jain, Trever Ball, Amparo Abel-Bey, Keisha Bowman, Shivanthidevi Gandhi, Pranisha Gautam-Goyal, Zenobia Brown

**Affiliations:** Northwell Health, New Hyde Park, New York; North Shore University Hospital, Manhasset, New York; North Shore University Hospital, Manhasset, New York; Northwell Health, New Hyde Park, New York; Northwell Health, New Hyde Park, New York; Northwell Health, New Hyde Park, New York; North Shore University Hospital, Manhasset, New York; Zucker School of Medicine at Hofstra/Northwell, Manhasset, New York; Northwell Health, New Hyde Park, New York

## Abstract

**Background:**

Immunocompromised patients are at high risk for COVID-19 morbidity and mortality. While findings from the PROVENT study led to emergency use authorization of tixagevimab/cilgavimab (TC) for pre-exposure prophylaxis, less is known of its protection in vaccinated immunocompromised patients against the SARS-CoV-2 Omicron variants and subvariants. The purpose of this study was to evaluate association between TC and self reported SARS-CoV-2 infection during the Omicron variant surge in vaccinated immunocompromised persons.

**Methods:**

This was a retrospective study that surveyed and evaluated electronic health records of patients who received TC between January 2022-November 2022. TC’s protection against SARS-CoV-2 infection was evaluated preliminarily by average monthly infection rates before and after the BA.5 variant was regionally predicted to be < 50% of infections (October 29, 2022). Multivariate logistic regression was used to determine odds of infections during 4 months before and 4 months after subvariants, while controlling for age, race, gender, vaccination status, and immunocompromised conditions.

**Results:**

43% (126/293) of recruited patients completed the study’s survey. All patients were vaccinated with at least primary series for SARS-CoV-2 infection. A majority were female (53.2%), > 65 yrs (61.1%), White (70.4%), and immunocompromised with solid malignancy (61.1%). Average monthly infection rate during the BA.5 variant was 4.4% and after was 12.1%. Patients who received a vaccine booster twice were 68% less likely to be infected prior to Omicron subvariants compared to those who only received their primary vaccination series (OR=0.32; p=0.10). During this period, patients with rheumatoid arthritis were 5.6 times more likely than patients with solid malignancy to be infected (OR=5.64; p=0.07). During Omicron subvariants, patients with Lupus were most likely to be infected compared to patients with solid malignancy, but not statistically significant (OR=1.78; p=0.69).

**Conclusion:**

In the presence of changing vaccine coverage, therapies, and changing variants, the effectiveness of TC in the Omicron era is difficult to assess. These preliminary findings describe a real world snapshot of SARS-CoV-2 infections in high-risk patients who received TC.

**Disclosures:**

**All Authors**: No reported disclosures

